# Reparations for Indigenous Peoples in the USA and Canada

**DOI:** 10.1371/journal.pgph.0006688

**Published:** 2026-06-17

**Authors:** Joseph P. Gone

**Affiliations:** 1 Department of Anthropology, Harvard University, Cambridge, Massachusetts, United States of America; 2 Department of Global Health and Social Medicine, Harvard Medical School, Boston, Massachusetts, United States of America

## Abstract

Indigenous populations comprise some 400 million people worldwide, hailing from 5,000 descent communities. Although contemporary Indigenous Peoples are exceedingly diverse, they frequently share brutal histories of colonization. These histories of subjugation by outsiders have yielded catastrophic impacts on Indigenous health and well-being, including marked inequities in health status. Specifically, Indigenous communities often suffer from disproportionately high rates of infant and maternal mortality, infectious diseases, parasite loads, diseases of forced acculturation (such as diabetes and hypertension), exposure to environmental contaminants, and stark anomic health conditions (such as addiction, violence, suicide). Even in the comparably wealthy nations of the USA and Canada, Indigenous health inequities persist. It is common to attribute Indigenous health inequities to poverty and despair; and yet, for most Indigenous Peoples, these disparities result from harms that were deliberately engineered by others. Repair for colonial legacies of poor health within Indigenous communities will require adequately funded healthcare, robust social services, and the eradication of poverty. Beyond this, reparations for Indigenous Peoples should include recognition of Indigenous sovereignty, honoring of historical treaties, and return of Indigenous lands.

## Author summary

Contemporary Indigenous populations number around 400 million people worldwide. Although Indigenous Peoples are exceedingly diverse, they frequently share histories of colonial subjugation by outsiders. Histories of colonial subjugation have yielded catastrophic impacts on Indigenous health and well-being. Although commonly attributed to poverty and despair, health inequities for most Indigenous communities typically resulted from harms engineered by others. Repair for colonial legacies of poor Indigenous health will require adequately funded healthcare, robust social services, eradication of poverty, recognition of Indigenous sovereignty, and return of Indigenous lands.

## Introduction

As part of the special section of this journal dedicated to reparations in global health, this article considers the case for redress for Indigenous Peoples based on the devastating health consequences of longstanding colonial subjugation. Although there is no uniform definition of *indigeneity*, Indigenous Peoples are all populations whose collective existence predates the establishment of nation-states in their territories and whose identities persist in association with identifiable locales [1]. Globally, Indigenous populations include an estimated 400 million people comprising some 5,000 descent communities today [2]. Official identification of Indigenous populations is complicated by disparate national policies concerning whether and how to designate specific descent communities as First Peoples. For example, the USA maintains government-to-government relations with 575 “federally recognized tribes,” while Rwanda has banned the use of the term “indigenous” to describe those of Twa (Pygmy) lineage.

Despite their long histories of marginalization and subjugation by outsiders, it is difficult to delineate shared attributes across diverse Indigenous populations. Indigeneity is routinely linked to brutal histories of colonization, enduring group identification, spiritual relationships to local ecologies, and other distinctive linguistic and cultural practices. Given long histories of marginalization and subjugation by outsiders, however, Indigenous Peoples have navigated their colonial status in a variety of ways. Moreover, one challenge to discussing Indigenous populations is the need to avoid essentializing rhetoric, romanticized tropes, or primitivist stereotypes about indigeneity and Indigenous Peoples’ relationships to place, land, and modernity (which Indigenous Peoples themselves sometimes strategically harness for political purposes in contexts of overwhelming marginality).

Representative and robust data concerning almost all facets of Indigenous Peoples’ lives are strikingly lacking [3], with the greatest availability of research found in wealthy countries with well-funded research infrastructures (e.g., Canada, Australia, New Zealand, and the USA, though even for American Indians there remain concerns about data genocide [4,5]). Nevertheless, Indigenous populations across the globe tend to share contemporary circumstances born of colonization. These histories include genocide, violence, dispossession, forced migration, cultural denigration, religious repression, coercive assimilation, rampant discrimination, bureaucratic control and neglect, and impoverishment.

## Indigenous health inequities

These depredations have had catastrophic impacts on Indigenous health and well-being. On almost any health indicator, Indigenous Peoples fare worse when compared with their surrounding national populations [1,2]. This trend holds true even in affluent nations such as the USA and Canada (ranked fifteenth and thirteenth, respectively, out of nearly 200 nations in the 2018 Human Development Index) [6]. Both countries harbor significant Indigenous populations, whose enduring privations amid great national wealth are striking when considering reparations for Indigenous Peoples.

In a series of articles dedicated to Indigenous health that appeared in 2006 [7–10], as well as two additional reviews that appeared in 2009 [11,12], health inequities were described for a wide range of conditions for Indigenous populations in different parts of the globe (Australia, Canada, New Zealand and across Latin America, and Africa). Commonly observed indicators of poor health include inequities in infant and maternal mortality, infectious diseases (e.g., HIV/AIDS, tuberculosis, Coronavirus), parasite loads, life expectancy at birth, diseases of forced acculturation or rapid transition (e.g., diabetes mellitus, cardiovascular disease, hypertension), exposure to environmental contaminants (e.g., chemicals from mining operations), and anomic health conditions associated with cultural dispossession, bleak economic prospects, and rampant demoralization (e.g., addiction, violence, suicide).

Even in the USA, the world’s largest economic power, Indigenous health inequities persist [13,14]. Some 9.7 million Americans self-identify as American Indian or Alaska Native (AIAN), representing 2.9 percent of the national population (and including ~3 million members of federally recognized tribes). The federally funded Indian Health Service (IHS) has reported numerous health disparities between AIANs and the “U.S. All Races” population. Specifically, AIANs exhibit lower life expectancy at birth (5.5 years lower) and higher mortality from the following causes: alcohol-induced death (6.6 times higher), chronic liver disease and cirrhosis (4.6 times higher), diabetes mellitus (3.2 times higher), unintentional injuries (2.5 times higher), assault/homicide (2.1 times higher), drug-induced death (1.8 times higher), influenza and pneumonia (1.8 times higher), intentional self-harm/suicide (1.7 times higher), septicemia (1.6 times higher), and kidney disease (1.5 times higher) [15]. The COVID-19 pandemic took a particularly devastating toll on AIAN communities, fueling alarming trends in reduced life expectancy [16,17].

In Canada, some 1.68 million people identify as Indigenous (around five percent of the national population), comprised of First Nations, Inuit, and Métis people with ancestral ties to roughly 630 Indigenous communities (about three-quarters of the self-identified First Nations population is registered as such in accordance with Canada’s Indian Act). Despite a comparatively high quality of life overall in Canada, Indigenous Canadians suffer from disproportionately high rates of health problems. These include HIV/AIDS, tuberculosis, diabetes mellitus, addiction, violence, and suicide [12,18]. For both the USA and Canada, patterns of health disparities underscore the scope of inequity in the life experiences of Indigenous People [14,19,20].

## Colonial origins of health inequities

It is common to attribute such health vulnerabilities—and ensuing inequities among Indigenous populations—to poverty and despair. However, it is also crucial to acknowledge that, for most Indigenous Peoples, these disparities result from harms that were deliberately engineered by others. The production of Indigenous disadvantage is far from accidental; it has historically involved processes that were intended and designed to dispossess, subjugate, and even exterminate. Indeed, even the epidemic diseases first brought by Europeans to North America would not have been so deadly had they not been accompanied by violent dispossession leading to compromised immune function within Indigenous populations [21]. Present-day Indigenous health inequities are the downstream outcomes of ruthless processes of colonization [12,20,22,23], leading to recognition of colonial determinants of health [24].

### Lakota history as case illustration

Consider the contemporary Lakota populations, many of whom reside on or near Indian reservations in North and South Dakota in the USA [25–27]. The Lakota signed a treaty with the U.S. federal government in 1851 that reserved large tracts of their traditional territory for their exclusive use. This territory included the Black Hills, a mountain range that remains sacred to the Lakota. Despite this agreement, settler colonists were allowed to migrate to this territory, especially after gold was discovered. The result was intermittent warfare between the Lakota and the US government throughout the latter half of the nineteenth century. Increasing pressure was put on the Lakota to withdraw to reservations. Those who resisted continued to fight the U.S. Army, leading to the annihilation of General Custer’s military command at the Battle of the Little Bighorn in 1876. In consequence, the U.S. Congress unilaterally seized the Black Hills and surrounding territory in violation of treaty.

Most reservations were controlled by White political appointees who wielded near dictatorial power in reservation affairs. Successive cohorts of Lakota children were compelled to attend government or missionary-administered industrial boarding schools devised under the slogan, “Kill the Indian, Save the Man” [28,29]. The boarding schools were frequently operated in military fashion, complete with uniforms and drilling, as well as daily labor, inadequate nutrition, harsh corporal punishment, and all-too-frequent physical and sexual assault by school staff. The despondency of reservation life led to a Lakota religious revitalization movement called the Ghost Dance. Practice of the Ghost Dance, which included a belief that White American domination would end, frightened settler-colonists adjacent to the reservations, leading to renewed military mobilizations against the Lakota to quell the movement.

On December 29, 1890, a band of Lakota were being disarmed by the U.S. Seventh Cavalry near Wounded Knee Creek in present-day South Dakota when a rifle discharged. In the resulting tumult, cavalry troopers fired cannons, killing between 150 and 400 Lakota, mostly noncombatants. Although Wounded Knee marked the cessation of warfare between the Lakota and the USA, the battle for the return of the Black Hills to Lakota control continued. Throughout much of the 20^th^ century, the Lakota filed lawsuits against the USA for theft of the Black Hills. Most of these suits were dismissed on grounds that the U.S. Congress wielded plenary power to dispossess or even terminate the rights of a conquered people.

### Recent Lakota resistance

In the 1970s, the Indigenous civil rights or “Red Power” movement orchestrated resistance to federal control and oversight, leading once again to eruptions of state violence [30]. In 1972, the American Indian Movement (AIM) organized the Trail of Broken Treaties, a caravan of Indigenous activists that drove to Washington, DC, to protest the treatment of Indigenous Peoples. They issued a Twenty-Point Position Paper demanding that the federal government uphold treaty rights, restore treaty-making, and return lands.

Soon thereafter, AIM seized buildings in the village of Wounded Knee on the Pine Ridge reservation, engaging in an armed standoff with federal troops while demanding that the federal government investigate treaty violations and federal corruption. In 1975, AIM activists killed two agents of the Federal Bureau of Investigation, which had targeted AIM in its secret domestic Counterintelligence Program [27,30]. Ultimately, the Lakota legal struggle finally gained traction: in 1980, the U.S. Supreme Court ruled that the Black Hills had been stolen by the United States and awarded the Lakota $105 million (USD) in compensation [25–27].

### Indigenous historical trauma

Lakota people today suffer from a host of health inequities (e.g., adverse childhood experiences, diabetes mellitus, addiction, posttraumatic stress disorder, suicide) [14]. Moreover, the counties that contain Lakota reservations are among the poorest in the nation. Indeed, these contemporary conditions—engineered through colonization of the Lakota by the USA—led to widespread adoption of the term *historical trauma* to explain the health inequities that Lakota populations experience [31,32]. Historical trauma refers to the collective, cumulative, and intergenerational impacts of colonial subjugation on Indigenous Peoples [23]. A key element of this conceptual framework is colonial anomie, which results from the rapid and profound social disruptions that are intrinsic to the experience of being colonized. Symptoms of colonial anomie include the communal loss of identity, orientation, purpose, and belonging; these are enacted and embodied as postcolonial disorders (e.g., addiction, suicide) [33,34].

Tracing an unambiguous chain of causality from specific forms of past colonial subjugation to contemporary population-based health inequities is challenging. However, widely shared patterns of health vulnerability across diverse Indigenous Peoples around the globe are revealing. For example, the experiences of the Inuit in the USA, Canada, and Greenland share a pattern in which sedentarization by government policy (which occurred at different historical moments in each of these national settings) was followed a generation later by an epidemic of youth suicide. The temporal link between sedentarization and suicide – despite occurring at different times in each nation’s history – implicates a coercive shift in lifestyle as a cause of disparate health outcomes [35].

## Indigenous reparations

Beyond such clearly designated policy impacts, experiences of racism, discrimination, and marginalization likely intersect with colonial subjugation to create embodied responses that subsequently compromise health [22,36–39]. What then might reparations look like for Indigenous Peoples? In the USA, a Court of Indian Claims operated for nearly four decades in pursuit of repair for stolen Indigenous lands, compensating hundreds of AIAN Tribal Nations with monetary awards [40], though there was no attention to the health impacts of this restitution. Another salient, recent example emerged from the Truth and Reconciliation Commission (TRC) of Canada. In 2015, Canada completed a national reconciliation process pertaining primarily to coercive assimilation—and legacies of child abuse and criminal sexual assault—wrought by its notorious system of residential schools for Indigenous children. Following years of soliciting testimonies from Indigenous survivors of the residential schools, the TRC released 94 recommendations in a document titled “Calls to Action,” making specific suggestions for addressing the legacies of colonization and taking steps toward reconciliation [41].

### Indigenous health reparations

The Legacy section of the TRC report included recommendations in the five domains of child welfare, education, language and culture, health, and justice. There were seven specific calls to action associated with health: acknowledgment of the harms wrought by Canadian policy, a commitment to remedy health inequities, recognition of the needs of Indigenous People who live away from reserved lands, proper funding for health services, affirmation of indigenous healing practices, Indigenous inclusion in the health professions, and mandatory education about Indigenous Peoples in health professional training [41]. In a recent assessment of Canadian progress on these Calls to Action, only 13 of 94 recommendations had been completed in the eight years since they were released, including none of the health recommendations [42].

Obviously, Indigenous reparations will at minimum entail a commitment by colonizing states to adequately resource health services for Indigenous communities. In the USA, many treaties with AIAN Tribal Nations promised government medical services in exchange for Indigenous lands. Indeed, AIAN treaty rights and the associated federal trust responsibility remain the foundation for modern-day health care for tribal communities. And yet, owing to its dependence on annual discretionary mechanisms for federal appropriations, the Indian Health Service is chronically underfunded at just 42 percent per capita of what the U.S. government expends on health care for other beneficiaries [43]. Adequately funded health care is a necessary form of repair owing to its direct effects on improving AIAN health, and yet more and better medical services are in themselves insufficient for ultimately resolving the ills of “Indian Country.”

### Beyond health reparations

Beyond health care, other government-funded, Indigenous-administered social services (e.g., child care, job training, supportive housing) are urgently needed. Moreover, additional efforts by settler colonial nation states must be made to eradicate dire poverty within Indigenous communities. Additional funding is only part of the solution, however. Currently, the settlement funds set aside for the Lakota from the historical court judgment pertaining to the theft of their land has amassed to over $1 billion (USD). The Lakota have refused to accept this payment, insisting instead on the return of the Black Hills to their sovereign jurisdiction [44,45]. The demands contained within AIM’s Twenty-Point Position Paper remain relevant today: the United States must recognize AIAN sovereignty, honor historical treaties, and return Indigenous lands.

Indigenous sovereignty is affirmed and supported through treaty rights, both of which pertain to Indigenous lands. Indeed, discursively expansive calls for decolonization in metaphorical fashion (e.g., decolonizing education and health care) might actually undermine Indigenous aspirations for resumption of territorial control and repatriation of lands [46]. Lands are community resources that can serve a variety of functions and purposes, but Indigenous “land back” movements increasingly emphasize land-based healing [47]. As one example, the Omushkego Cree of subarctic Ontario have undertaken a series of land-based approaches to health and well-being that involve summer programs for youth in which culturally knowledgeable elders guide them in revitalizing traditional hunting, harvesting, and fishing practices [48].

By deliberately connecting Cree elders and youth out on their traditional lands, these seasonal programs facilitate youth knowledge, identity, well-being, and healing. Participants reported positive emotions, intergenerational knowledge transfer, greater cultural continuity, and strengthened social and community networks through relating with each other on the land and, indeed, cultivating a relationship with the land [48]. Thus, the significance of land back for Indigenous communities extends well beyond economic considerations, which explains the Lakota refusal to accept substantial settlement funds for the theft of their sacred Black Hills. In the end, with respect to their long-term well-being, the return of the Black Hills—much of which is still held by the U.S. federal government—may be the single most effective health intervention imaginable for the Lakota people. Similar reparative efforts stand to improve the health status of Indigenous Peoples throughout the world.

## Conclusion

Indigenous Peoples around the globe remain heirs to a shocking legacy of compromised health. This inequitable health burden arises primarily from long histories of colonial subjugation that desperately require international attention toward acknowledgment, recognition, redress, and repair by relevant nation-states. Beyond investments in health and social services, as well as in the quality of Indigenous life and livelihood, reparations will also entail renewed regard for Indigenous sovereignty and resolute restoration of Indigenous lands.
